# Phenotypic variation in metabolism and morphology correlating with animal swimming activity in the wild: relevance for the OCLTT (oxygen- and capacity-limitation of thermal tolerance), allocation and performance models

**DOI:** 10.1093/conphys/cov055

**Published:** 2016-01-11

**Authors:** Henrik Baktoft, Lene Jacobsen, Christian Skov, Anders Koed, Niels Jepsen, Søren Berg, Mikkel Boel, Kim Aarestrup, Jon C. Svendsen

**Affiliations:** 1 National Institute of Aquatic Resources, Technical University of Denmark, Silkeborg, Denmark; 2 Interdisciplinary Centre of Marine and Environmental Research, University of Porto, Porto, Portugal; 3 National Institute of Aquatic Resources, Technical University of Denmark, Charlottenlund, Denmark

**Keywords:** Aerobic metabolic scope, fineness ratio, morphology, OCLTT hypothesis, performance and allocation models, standard metabolic rate

## Abstract

Ongoing climate change is affecting animal physiology in many parts of the world. Using metabolism, the oxygen- and capacity-limitation of thermal tolerance (OCLTT) hypothesis provides a tool to predict the responses of ectothermic animals to variation in temperature, oxygen availability and pH in the aquatic environment. The hypothesis remains controversial, however, and has been questioned in several studies. A positive relationship between aerobic metabolic scope and animal activity would be consistent with the OCLTT but has rarely been tested. Moreover, the performance model and the allocation model predict positive and negative relationships, respectively, between standard metabolic rate and activity. Finally, animal activity could be affected by individual morphology because of covariation with cost of transport. Therefore, we hypothesized that individual variation in activity is correlated with variation in metabolism and morphology. To test this prediction, we captured 23 wild European perch (*Perca fluviatilis*) in a lake, tagged them with telemetry transmitters, measured standard and maximal metabolic rates, aerobic metabolic scope and fineness ratio and returned the fish to the lake to quantify individual *in situ* activity levels. Metabolic rates were measured using intermittent flow respirometry, whereas the activity assay involved high-resolution telemetry providing positions every 30 s over 12 days. We found no correlation between individual metabolic traits and activity, whereas individual fineness ratio correlated with activity. Independent of body length, and consistent with physics theory, slender fish maintained faster mean and maximal swimming speeds, but this variation did not result in a larger area (in square metres) explored per 24 h. Testing assumptions and predictions of recent conceptual models, our study indicates that individual metabolism is not a strong determinant of animal activity, in contrast to individual morphology, which is correlated with *in situ* activity patterns.

## Abstract (precis)

Combining physiological and morphological measures in the laboratory with registrations of detailed measures of field activity, we tested the hypothesis that individual activity patterns correlate with individual metabolism and morphology as proposed by several conceptual models. We found no evidence indicating an effect of metabolism, whereas morphology correlated with several activity measures.

## Introduction

Aerobic metabolism in animals is dependent on several abiotic factors, including ambient water temperature and CO_2_ and O_2_ levels. For example, metabolism associated with maintenance increases as a function of temperature in ectothermic animals (Brett, 1964; Tirsgaard *et al.*, 2015). Consequently, metabolic performance, typically measured as oxygen consumption rate, has been identified as a key component in predicting the reaction of aquatic ectothermic animals to climate change and ultimately their conservation through the oxygen- and capacity-limited thermal tolerance (OCLTT) hypothesis (Pörtner, 2010). Specifically, the OCLTT model proposes that the functional capacity of systems supplying and using oxygen sustains the aerobic performance capacity of the organism (Pörtner, 2010) and becomes limiting at high temperature extremes (Bozinovic and Pörtner, 2015). The OCLTT represents a major tool to predict the consequences of variation in temperature, oxygen availability and pH in the aquatic environment within the emerging field of conservation physiology. A central tenet of the OCLTT hypothesis is that aerobic metabolic scope (AMS) is a principal physiological trait governing many other performance traits (e.g. growth, digestion, reproduction, immune function, muscular activity and thermal tolerance). Aerobic metabolic scope is defined as the excess oxygen available above oxygen demand for maintenance and fuels the performance capacity of the animal (Pörtner and Lannig, 2009), which is therefore limited by the maximal aerobic metabolic rate. Although several recent studies question the broad applicability of the OCLTT hypothesis (e.g. Clark *et al.* 2013; Ern *et al.* 2014; Norin *et al.* 2014; Wang *et al.* 2014), it is conceivable that the link between AMS and performance, as suggested by the OCLTT hypothesis, should be revealed by positive correlations between individual AMS and activity levels. This assumes that individual activity levels exhibit periodic or frequent elevations close to the performance ceiling or that maximal activity levels consistently correspond to a certain fraction of the performance ceiling. Correlations between individual AMS and activity levels have, however, rarely been tested in the wild.

A link between consistent individuality in metabolism and activity has also been suggested by recent studies unrelated to the OCLTT hypothesis (Careau *et al.*, 2008; Biro and Stamps, 2010; Burton *et al.*, 2011; Mathot and Dingemanse, 2015). A mechanism proposed to facilitate this coupling is the concept of an individually sized ‘metabolic machinery’ that on the one hand enables energy output, but on the other hand requires maintenance (the ‘performance model’ *sensu* Careau *et al.* 2008). Following this, individuals with relatively large machinery capable of producing more energy to fuel activities (e.g. movement, generation of somatic or gonadal tissue) are faced with a need for a higher energy uptake. For example, as individuals are expected to display behaviour that increase food intake rate, ‘high-energy’ individuals should be more active, be bolder and explore larger areas to sustain their metabolic machinery, assuming they rely on an active food search strategy. In contrast, ‘low-energy’ individuals will have lower amounts of available energy for activity but also have lower maintenance needs, i.e. a lower need to be active. Thus, the performance model predicts a positive relationship between standard metabolic rate (SMR) and activity. Alternatively, a coupling between metabolic traits and activity could exist according to the ‘allocation model’ (*sensu* Careau *et al.* 2008), in which an individual animal has a limited amount of energy to allocate between SMR and activity. Individuals with a need for more energy to maintain SMR can allocate less energy to activity than individuals with lower SMR. In contrast to the performance model, therefore, the allocation model predicts a negative relationship between SMR and activity. Regardless of the complex mechanisms linking metabolism, thermal tolerance and animal activity (Horodysky *et al.*, 2015), the examination of related correlations is a useful approach to examine conceptual models and their assumptions and predictions.

Interspecific differences in morphology of fish are known to reflect differences in swimming capabilities and general behaviour. For instance, the posterior positioning of the dorsal and anal fins in northern pike (*Esox lucius*) reflects an adaptation to sprint-based foraging (Craig, 1996), whereas thunniform body shapes are optimal for cruising (Webb, 1984). Likewise, intraspecific individual variation in body shape could affect the cost of transport and translate into behavioural variation. For fish moving through water, overall body shape is a major determinant of resistive drag and thereby cost of transport. Combining physics theory and hydrodynamic modelling, fish body form may be simplified to a prolate spheroid and described by the fineness ratio (FR), defined as length divided by maximal diameter (i.e. body depth in most fish species). From such modelling, FRs ranging between 4.5 and 8 have been shown to be most efficient (Blake, 1983; Chung, 2009; Langerhans and Reznick, 2010). Given that an optimum exists, it is conceivable that individual morphological differences will influence cost of transport and, subsequently, behaviour. Using swimming respirometry, Ohlberger *et al.* (2006) found a relationship between swimming costs and FR in common carp (*Cyprinus carpio*) and roach (*Rutilus rutilus*). Boily and Magnan (2002) found higher swimming costs for stout than slender individuals of brook char (*Salvelinus fontinalis*) and yellow perch (*Perca flavescens*); however, the authors did not report FR for the fish. Using telemetry in the field, Hanson *et al.* (2007) found that body shape influenced mean swimming speeds of largemouth bass (*Micropterus salmoides*).

Most studies linking metabolic and morphological traits to activity levels and behaviour have been performed in laboratory settings. However, several studies related to metabolism and behaviour suggest that care should be taken when extrapolating laboratory findings to natural settings (Blake, 1991; Klefoth *et al.*, 2012; Vanin *et al.*, 2012; Fisher *et al.*, 2015). For instance, fish behaviour in laboratory trials might not be a good predictor for fish behaviour in the wild, as indicated by Klefoth *et al.* (2012). Thus, although correlations between individual metabolism and behaviour are found in laboratory experiments, it is largely unknown whether such correlations exist in nature. Recent technological advents have enabled field studies on detailed *in situ* fish behaviour (Lucas and Baras, 2000; Cooke *et al.*, 2005; Svendsen *et al.*, 2011; Baktoft *et al.*, 2015), thereby facilitating the inclusion of volitional behaviour of free-swimming animals in this research area.

In the present study, we tested the overarching hypotheses that individual variation in metabolic and morphological traits influence and, consequently, are correlated with *in situ* behavioural variation. Specifically, we examined predictions derived from the OCLTT hypothesis, the allocation model and the performance model that fish activity levels would correlate with metabolic physiology measured as AMS and SMR. Additionally, we examined the prediction that morphology measured as FR would be correlated with fish activity levels. To this end, we captured European perch (*Perca fluviatilis*) to quantify their physiology and morphology in the laboratory and returned the fish to their natal lake, where we monitored their activity patterns using high-resolution positional telemetry.

## Materials and methods

### Fish

Twenty-three European perch [mean body mass 54.2 ± 15.3 g (mean ± SD) and fork length (FL) 16.4 ± 1.4 cm (mean ± SD), range 14.1–19.7 cm] were captured by angling in a lake (Lake Gosmer; 55°55′42N, 10°10′50E; 1 ha; Baktoft *et al.* 2015) and transferred to the laboratory in mid-September 2010. Fish were kept in flow-through tanks (3 m × 3 m), maintained at 16 ± 1°C and fed daily with roach (*Rutilus rutilus*). Light regimen was 14 h–10 h (light–dark). Each fish was anaesthetized (benzocaine 300 ppm) and tagged with an acoustic transmitter (Lotek MAP 6_2, burst interval 30 s, 15 mm × 6.2 mm, 1.1 g in air) and a passive integrated transponder tag (12 mm × ’2.12 mm; 95 mg in air; Loligo Systems, Tjele, Denmark) for rapid identification. Both tags were inserted through an incision in the body cavity and closed with a single absorbable suture (Vicryl 5-0FS-2; Ethicon, Piscataway, NJ, USA) following standard procedures (e.g. Boel *et al.*, 2014; Jacobsen *et al.*, 2014). An experienced fish surgeon (Cooke *et al.*, 2003) performed surgical implants in accordance with the guidelines described in permission 2012-DY-2934-00007 from the Danish Experimental Animal Committee. After surgery and recovery (10–20 min), all fish were returned to their holding tank. Fish were then allowed a 10 day period to recover from the surgical procedure (e.g. wound healing) and to acclimate to the laboratory holding facilities before quantification of metabolism and morphology. Fish were isolated and unfed for 24 h before data collection as detailed below. During the holding period in the laboratory (19 days), all fish chased and consumed prey, incisions healed well, and sutures became partly absorbed. Overall fish condition seemed good, although caudal fins were slightly eroded.

### Metabolism

Four acrylic respirometer chambers (each 0.54 l) were used concurrently for simultaneous measurements on four fish. Chambers were submerged in aerated water (>95% air saturation) drawn from the recirculating system, i.e. the same water as used for fish holding. Water temperature was kept at 16°C (range 15.9–16.1°C) using a temperature-controlling instrument (TMP-REG; Loligo Systems).

Measurements of oxygen consumption rate (${\dot{M}}_{O_{2}}$; in milligrams of oxygen per kilogram per hour) were carried out using intermittent flow respirometry (Forstner, 1983; Rosewarne *et al.*, 2015). Each respirometer chamber was fitted with two inlet and two outlet ports and two water pumps (Svendsen *et al.*, 2012; Tirsgaard *et al.*, 2015). Fish did not display rheotaxic responses to chamber flow and remained stationary and calm, apart from intermittent movements. Oxygen partial pressure (in kilopascals) was measured inside the respirometers at 1 Hz using galvanic oxygen sensors (Mini DO Probe; Loligo Systems). Oxygen levels ≥80% air saturation in the respirometers were secured using flush pumps that were activated intermittently and controlled by AutoResp software (Loligo Systems). Between flushings, the declining oxygen partial pressure was recorded to calculate ${\dot{M}}_{O_{2}}$ using the following equation:

$${\dot{M}}_{O_{2}}=K\times V\times \beta \times M^{-1},$$

where *K* is the linear rate of decline (in kilopascals per hour) in the oxygen content over time (in hours) in the respirometer, *V* is the volume of the respirometer (in litres) corrected for the volume of fish, β is the solubility of oxygen in the water (in milligrams of oxygen per litre per kilopascal; β = 0.4755) and *M* is the body mass of the fish (in kilograms). The coefficient of determination (*r*^2^) associated with each ${\dot{M}}_{O_{2}}$ measurement was always ≥0.90, similar to previous studies (Genz *et al.*, 2013; Svendsen *et al.*, 2013). Corrections of background respiration (i.e. microbial respiration) followed Rosewarne *et al.* (2015).

Maximal metabolic rate (MMR) was elicited using the chase protocol described previously (Cutts *et al.*, 2002; Norin and Malte, 2011; Svendsen *et al.*, 2014). Individual fish were transferred to a circular trough and chased manually until exhaustion as evidenced by the fish not reacting to being turned upside down and lifted partly out of the water. Chasing lasted for ∼5 min. When exhausted, fish were immediately transferred to the respirometer, and measuring of metabolism commenced within 10 s. The MMR was the highest of three consecutive ${\dot{M}}_{O_{2}}$ measurements (Svendsen *et al.*, 2012). It is unlikely that MMR can be sustained beyond a relatively short period (<1 h). In fish, this is probably because of the high ion flux at the gills that is inevitably associated with high or maximal oxygen uptake at the gills.

**Figure 1:**
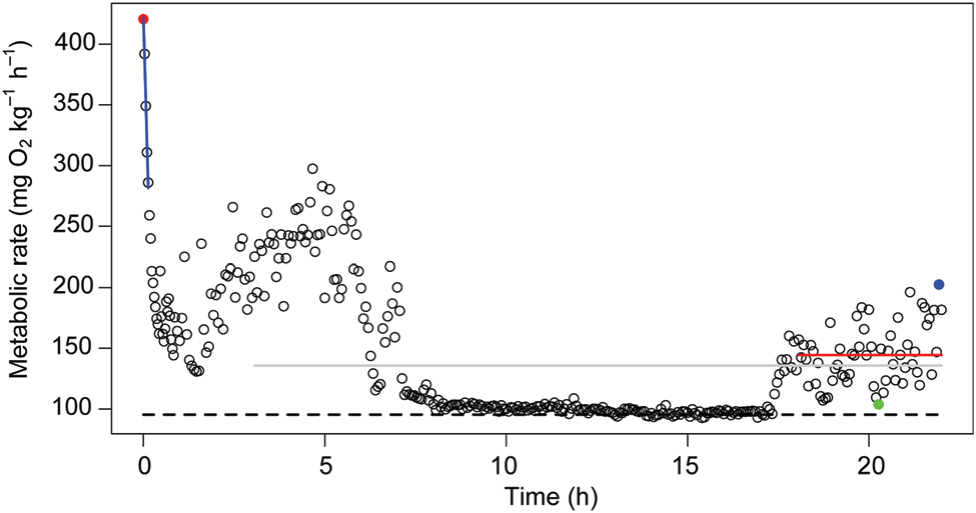
Metabolic Q12 rate (${\dot{M}}_{O_{2}}$; in milligrams of oxygen per kilogram per hour) over time (in hours) in European perch (*Perca fluviatilis*) measured using intermittent flow respirometry. A chase protocol was applied to elicit maximal metabolic rate (MMR; red datum), followed by declining ${\dot{M}}_{O_{2}}$ values. A linear regression line was fitted to the first five data points recorded immediately after the chase protocol (blue line), and the slope was used to estimate the rate of recovery. Standard metabolic rate (SMR) was estimated as the average of the lowest 10th percentile of all ${\dot{M}}_{O_{2}}$ measurements throughout the period of data collection (22 h; dashed line). After 3 h of acclimation, the average ${\dot{M}}_{O_{2}}$ was recorded over 19 h (grey line). Routine ${\dot{M}}_{O_{2}}$ was recorded as the mean ${\dot{M}}_{O_{2}}$ during the last 4 h of respirometer confinement (red line). Likewise, the spontaneous minimal (green datum) and maximal (blue datum) ${\dot{M}}_{O_{2}}$ values were measured during the last 4 h of respirometer confinement. Aerobic metabolic scope (AMS) was estimated as the difference between MMR and SMR (i.e. red datum and dashed line), whereas spontaneous aerobic metabolic scope (in milligrams of oxygen per kilogram per hour) was estimated as the difference between the spontaneous minimal and maximal ${\dot{M}}_{O_{2}}$ (i.e. between the green and blue data points).

For each trial, the chase protocol ended around 14.00 h, and fish were left in the chambers for the following 22 h (Fig. 1). During this time, each fish was visually shielded using vertical opaque acrylic screens. Additionally, the entire set-up was shrouded in curtains to exclude visual disturbance of the fish. Pilot experiments showed that European perch ${\dot{M}}_{O_{2}}$ generally reached a stable low level within 60 min after the light went off. After each trial, all equipment was disassembled, disinfected and thoroughly rinsed.

Standard metabolic rate was estimated as the average of the lowest 10th percentile of all ${\dot{M}}_{O_{2}}$ measurements within the 22 h of respirometer confinement (Killen *et al.*, 2012b). Aerobic metabolic scope was calculated as the difference between MMR and SMR. In addition to MMR, SMR and AMS, this study quantified the following seven metabolic variables for each individual fish (Fig. 1): (i) rate of recovery was estimated as the slope of the relationship between time (in hours) and the five consecutive measurements of metabolic rate that were recorded immediately after the chase protocol; (ii) average ${\dot{M}}_{O_{2}}$ was recorded as the mean ${\dot{M}}_{O_{2}}$ during the last 19 h of data collection; (iii) metabolic variability was estimated as the standard deviation of all ${\dot{M}}_{O_{2}}$ measurements during the last 19 h of data collection (Careau *et al.*, 2008); (iv) routine ${\dot{M}}_{O_{2}}$ was estimated as the average ${\dot{M}}_{O_{2}}$ during the last 4 h of respirometer confinement (Killen *et al.*, 2012a); (v) spontaneous minimal ${\dot{M}}_{O_{2}}$ and (vi) spontaneous maximal ${\dot{M}}_{O_{2}}$ were estimated as the minimal and maximal values, respectively, during the last 4 h of respirometer confinement; and (vii) spontaneous AMS was estimated as the difference between (v and vi).

For all estimates of metabolism, body mass and metabolic rates were log_10_-transformed prior to analyses to normalize and linearize the data (Auer *et al.*, 2015). Residuals generated from each of these analyses differentiated those individuals having higher than expected metabolism for their body size (i.e. positive residuals) from those having metabolic rates lower than expected (i.e. negative residuals). Given that body mass can inﬂuence both metabolism and activity patterns, these estimates of mass-independent metabolic rates were used in subsequent analyses (Robertsen *et al.*, 2014). No body mass correction was applied for metabolic variables (i) and (iii) (i.e. rate of recovery and metabolic variability).

The SMR, MMR and AMS were analysed separately in subsequent analyses, and the dimensions of the data set containing the remaining seven metabolic metrics were reduced using a principal component analysis (PCA). Prior to the PCA, data were centred and scaled to have unit variance. The first three axes from the PCA (MET1, MET2 and MET3) were used as explanatory variables in subsequent analyses.

### Morphology

In addition to measuring FL, FR was calculated following Blake (1983) as FR = FL/maximal body depth. Fineness ratio is a dimensionless measure of overall body shape, in which low and high values indicate stout and slender individuals, respectively.

### Activity measures

**Figure 2:**
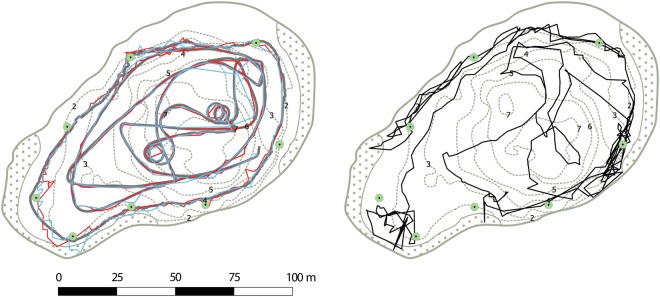
Overview of study lake, telemetry system and example data from test tracks (left) and a 24 h track from a single fish used in the study (right). Green circles indicate positions of the eight hydrophones; dotted areas show the extent of emergent macrophytes (*Tyhpa latifolia*); and dotted lines are depth isopleths, with depth given in metres. Transmitters surgically implanted in the fish emit acoustic signals. When these signals are detected on more than three hydrophones, a position can be calculated using hyperbolic multilateration based on time differences of arrival on each hydrophone (see Baktoft *et al.* 2015 and references therein for further details). Test tracks (left) were obtained by moving two transmitters (blue and red lines) attached to a vertical rod mounted on a boat. True trajectory of the test track (thick grey line) was obtained by a hand-held differential GPS unit held directly above the transmitters.

The European perch were returned to their natal lake upon completion of the laboratory protocol (all fish were returned on the same day). An acoustic positional telemetry system was used to record volitional *in situ* behaviour in Lake Gosmer (Fig. 2). In short, the telemetry system enabled near-continuous monitoring of tagged fish with high temporal and spatial resolution by yielding time-stamped geographical coordinates (mean precision 0.2 m; Baktoft *et al.*, 2015). From these data, we calculated the following: (i) daily individual movement as total moved distance in 24 h (*A*_day_; in metres per 24 h); (ii) instantaneous individual swimming speed (*U*_inst_; in metres per second); (iii) daily maximal individual swimming speed (*U*_max_; in metres per second); and (iv) daily covered area (AR_day_; in square metres per 24 h). For all analyses the complete data set was used, but note the selection criteria for swimming speed calculations given below. The *A*_day_ was calculated as the daily sum of Euclidian distances between consecutive positions. Swimming speeds (i.e. *U*_inst_ and *U*_max_) were calculated as the two-dimensional Euclidian distance between two consecutive points divided by change in time. Only instances with maximal obtainable temporal resolution between two registrations, i.e. the transmitter burst interval (=30 s), were used for estimates of swimming speed. Furthermore, only instances where the individual European perch were active were used for the measures of *U*_inst_ and *U*_max_. In order to identify these data objectively, we employed a hidden Markov model with location and level of activity as hidden states (Pedersen *et al.*, 2011), providing a measure of activity expressed as the probability that a given fish was active at a given time (*p*_active_). Only observations with *p*_active_ ≥ 0.75 were included in analyses of *U*_inst_. The *U*_max_ was defined as the individual daily maximum of *U*_inst_. Finally, the *AR*_day_ was estimated as the area of a 0.5 m buffer applied to daily tracks obtained by connecting consecutive positions with straight lines (Svendsen *et al.*, 2011). Overlapping daily buffers within an individual were merged before area calculation. The *AR*_day_ represents a measure of exploratory tendency, which may differ from *A*_day_ and estimates of swimming speed (Cote *et al.*, 2010; Svendsen *et al.*, 2011). The first 7 days in the lake were excluded to allow the European perch to re-acclimatize to their natural environment. All calculated activity measures were based on data obtained from the following 12 days, during which the lake was left undisturbed. Dissolved oxygen and water temperature were monitored using four optical probes (FDO 700 IQ; WTW, Weilheim, Germany) positioned 1.0, 2.5, 4.0 and 5.5 m below the surface.

### Statistical analysis

Random intercept linear mixed effects models were applied to assess whether individual activity in the lake could be explained by metabolic traits and/or morphology. In addition to FL, the following explanatory variables (*X*) were tested: FR, SMR, MMR, AMS, MET1, MET2 and MET3. As several of these explanatory variables were correlated, separate models were fitted for each of these to avoid collinearity in the models. Additionally, each activity measure (*A*_day_, *U*_inst_, *U*_max_ and *AR*_day_) was analysed separately using the same initial model:

$$\begin{array}{l} Y_{ijkp}=\alpha_{kp}+FL_{j}+X_{jp}+a_{jkp}+\epsilon_{ijkp}\\ \;\;\;\;\;\;\;\;\;\;\;\;\;\;\;a_{jkp}\tilde{}N(0,{\sigma_{akp}}^{2})\\ \;\;\;\;\;\;\;\;\;\;\;\;\;\;\;\epsilon_{ijkp}\tilde{}N(0,{\sigma_{jkp}}^{2}), \end{array}$$

in which observation *i* of activity measure *k* in fish *j* modelled by explanatory variable *p* equals the sum of a *kp*-specific common intercept (α*_kp_*), estimated effects of FL, the focal explanatory variable *p* of fish *j* (*X_jp_*), a *kp*-specific random intercept (*a_jkp_*) and *kp*-specific residual variation (ϵ*_ijkp_*). The random intercepts were assumed to be normally distributed with mean zero and *kp*-specific variances ${\sigma_{akp}}^{2}$. Additionally, residual variation in each model was assumed to be normally distributed with mean zero and variances ${\sigma_{jkp}}^{2}$ varying with activity measure *k*, explanatory variable *p* and fish *j*. To identify the parsimonious model best explaining the variation in each activity measure, initial models were compared with nested models excluding FL and *X* using Akaike information criterion (AIC) and likelihood ratio tests (Zuur *et al.*, 2009). The determined optimal models for each activity measure were further analysed to obtain parameter estimates and significance levels of FL and explanatory variable *X* where relevant. Significance tests were based on likelihood ratio tests using maximum likelihood estimation, whereas parameter estimates were obtained using restricted maximum likelihood estimation. The value of *U*_inst_ was log(*y* + 0.1)-transformed to meet model assumption of normality. Model validation based on informal visual inspection of plots of normalized residuals following Zuur *et al.* (2009) showed no signs of violation of model assumptions. To assess individual consistency of the activity measures, the intraclass correlation coefficients (ICCs) were calculated based on the optimal model for each activity measure (Zuur *et al.*, 2009). The variance structure allowing different variances for each fish necessitated that ICC was calculated for each fish separately, thus: $\mathrm{IC}C_{jkp}={\sigma_{akp}}^{2}/({\sigma_{akp}}^{2}+{\sigma_{jkp}}^{2})$.

All statistical analysis were done in R version 3.0.2 (R Core Team, 2013) using the nlme-package version 3.1-111 (Pinheiro *et al.*, 2013) in addition to base R functions.

## Results

### *In situ* activity

Of the 23 fish returned to the lake, three were consumed by northern pike naturally occurring in the lake and tagged as part of other studies (Baktoft *et al.*, 2013, 2012; Jacobsen *et al.*, 2014). Predation was identified when tracks of tagged European perch and northern pike were merged over several days. Additionally, four transmitters malfunctioned (i.e. no signals were received after release in the lake), leaving 16 fish to be included in analyses. During the tracking period (14–25 October, both days included), which coincided with the autumn turnover in the lake, mean water temperature was 9.1°C (range 7.7–11.4) and mean dissolved oxygen content was 5.9 mg l^−1^ (4.5–6.8).

**Figure 3:**
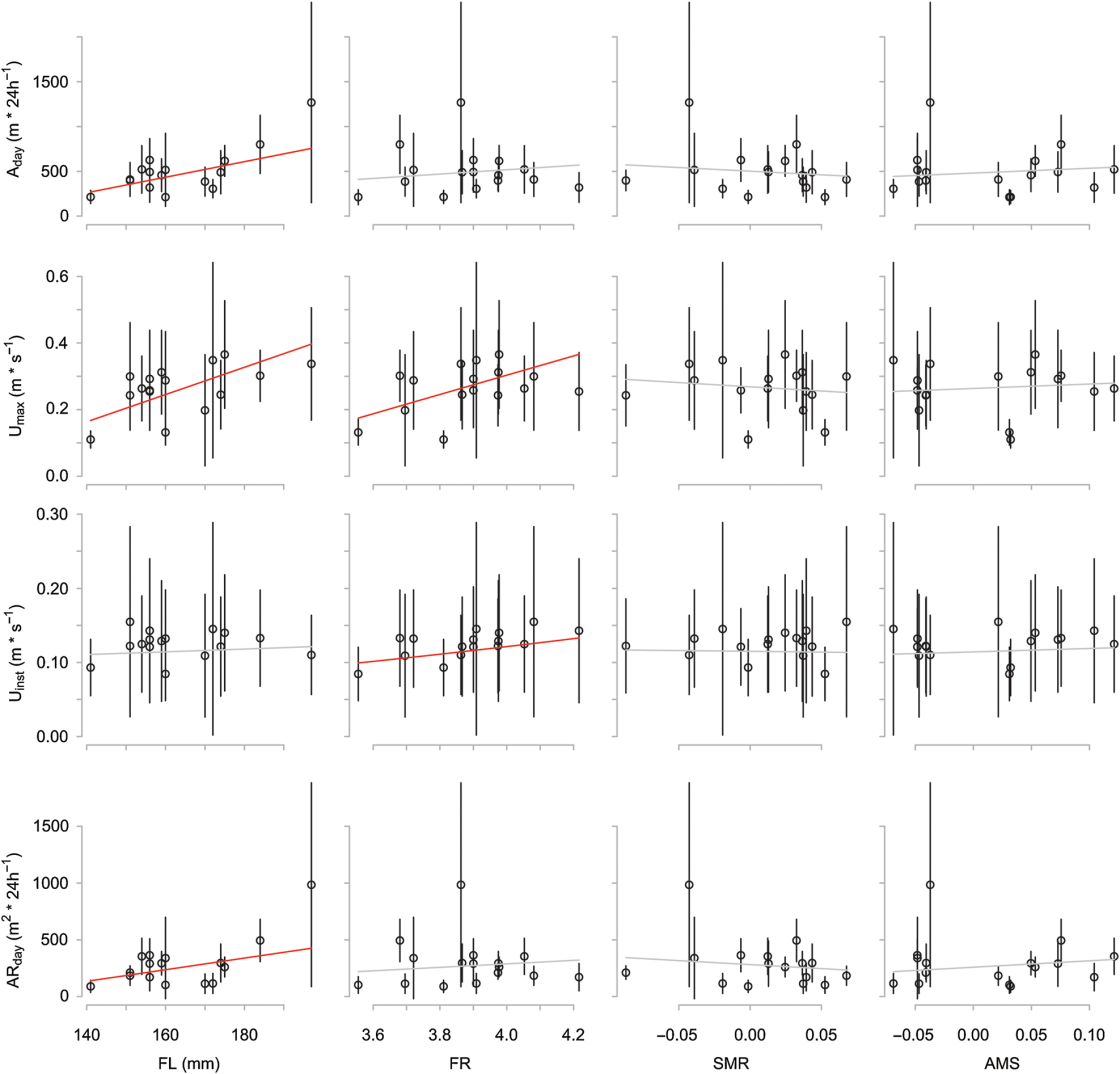
Visualization of raw data (mean values ± SD) and regression lines obtained from the random intercept linear mixed effects models. Significant regression lines (*P* < 0.05) are shown in red; non-significant in grey. Fork length (FL) and fineness ratio (FR) correlated positively with one or more of the activity measures. None of the metabolic measures [standard metabolic rate (SMR), maximum metabolic rate (MMR), aerobic metabolic scope (AMS), PCA axes representing other metabolic metrics (MET1–3)] was found to explain a significant amount of variation (Table 2; only SMR and AMS are shown in the figure). Metabolic measures were estimated by respirometry and corrected for body mass variation using analysis of residuals when appropriate.

Generally, recorded behaviour was variable both within and between individual fish (Fig. 3). Nevertheless, overall individual daily activity levels were partly repeatable over time, as indicated by the relatively high median ICCs found for *A*_day_ (median ICC = 0.34) and AR_day_ (median ICC = 0.49). This suggests that individual fish displayed consistent daily routines. However, the degree of this was highly variable between fish, as some individuals were very consistent whereas others showed virtually no day-to-day consistency (for example, AR_day_ individual ICC ranged from 0.01 to 0.81). By comparison, the variation in swimming speeds (*U*_max_ and *U*_inst_) was considerable both within and between individuals (median ICCs 0.051 and 0.037, respectively), although a few individual fish displayed highly consistent velocities (maximal individual ICC = 0.55).

### Individual metabolism is not correlated ’with activity

The first three axes from the PCA (MET1, MET2 and MET3) explained 92.7% of the variation in the data constituted by the seven metabolic measures obtained besides SMR, MMR and AMS (Table 1). The statistical models gave no indications suggesting that the metabolic traits influenced *in situ* activity patterns. Neither SMR, MMR and AMS nor the three PCA axes (MET1, MET2 and MET3) correlated significantly with any of the activity metrics (all *P*-values > 0.1; Table 2). Consequently, we found no support for either the allocation model or the performance model, because none of the activity metrics correlated with SMR. Likewise, we found no support for the hypothesis derived from the OCLTT model, because AMS did not correlate with the activity metrics (*P* > 0.1; Table 2). Mean values ± SD of SMR, MMR and AMS were 79.0 ± 9.9, 392.8 ± 61.1 and 313.8 ± 58.9 mg O_2_ kg^−1^ h^−1^, respectively.

**Table 1:** Results from the principal component analysis applied on seven metabolic measures

Metabolic measure	MET1	MET2	MET3
Rate of recovery	−0.24	0.19	0.84
Average ${\dot{M}}_{O_{2}}$	0.43	0.34	0.14
Metabolic variability	0.38	−0.02	0.44
Routine ${\dot{M}}_{O_{2}}$	0.45	0.23	0.05
Spontaneous minimal ${\dot{M}}_{O_{2}}$	0.35	0.52	−0.27
Spontaneous maximal ${\dot{M}}_{O_{2}}$	0.41	−0.42	0.06
Spontaneous AMS	0.35	−0.59	0.07
Cumulative variance explained	61.6%	79.8%	92.7%

Abbreviations: FL, fork length; FR, fineness ratio; SMR, standard metabolic rate; MMR, maximum metabolic rate; AMS, aerobic metabolic scope; MET1–3, PCA axes representing other metabolic metrics.

The cumulative variance explained by these three axes was 92.7%. Metabolic measures are explained in detail in the text. Abbreviations: AMS, aerobic metabolic scope; and ${\dot{M}}_{O_{2}}$, metabolic rate.

### Individual morphology is correlated ’with activity

Individual morphology correlated significantly with all four activity metrics (Fig. 3 and Tables 2 and 3). Fork length correlated positively with both *A*_day_ and AR_day_. Additionally, the optimal model for *U*_max_ found positive significant effects of both FL and FR. Finally, FR but not FL correlated positively with *U*_inst_. These findings indicate that inter-individual variation in swimming activity may be partly predicted by individual morphology. Additionally, body morphology favourable for higher swimming speed (i.e. higher FR) did not result in a larger searched area per 24 h (Fig. 2). Mean ± SD FR was 5.9 ± 0.3.

**Table 2:** Model comparisons for the four activity metrics modelled as function of FL*_j_* and *X_p_*

			*Y_k_* = *A*_day_		*Y_k_* = *U*_max_		*Y_k_* = *U*_inst_		*Y_k_* = AR_day_	
Model	FL*_j_*	*X_p_*	*P*-value	ΔAIC	*P*-value	ΔAIC	*P*-value	ΔAIC	*P*-value	ΔAIC
M0	**−**	**−**	n.a.	5.0	n.a.	13.1	n.a.	1.9	n.a.	1.9
M1	**+**	**−**	0.0083*	0*	0.026	10.1	0.94	3.9	0.0497*	0*
M1a	+	FR*_j_*	0.24	0.6	0.0005*	0*	0.015*	0*	0.37	1.2
M1b	+	SMR*_j_*	0.40	1.3	0.50	11.6	0.80	5.8	0.39	1.2
M1c	+	MMR*_j_*	0.47	1.5	0.76	12.0	0.45	5.3	0.32	1.0
M1d	+	AMS*_j_*	0.35	1.1	0.65	11.9	0.38	5.1	0.21	0.4
M1e	+	MET1*_j_*	0.59	1.7	0.88	12.1	0.65	5.7	0.20	0.4
M1f	+	MET2*_j_*	0.86	2.0	0.95	12.1	0.72	5.8	0.72	1.9
M1g	+	MET3*_j_*	0.30	0.9	0.74	12.0	0.33	4.9	0.21	0.4

Asterisks indicate optimal models. *P*-values represent the significance of the respective term tested with each model (i.e. FL*_j_* in M1 and *X_p_* in M1a–M1g) obtained using likelihood ratio tests. The ΔAICs were obtained by comparing each model with the optimal model for each respective activity measure.

**Table 3:** Summaries of optimal models for each activity measure

	Parameter	Estimate	SEM	*P*-value
*Y_k_* = *A*_day_	Optimal model:	M1: *A*_day_ = α*_kp_* + FL*_j_* + *a_jkp_* + ϵ*_ijkp_*		
	*α_kp_*	−954.1	519.3	0.066
	FL*_j_*	8.7	3.2	0.0083
	σ*_akp_*	132.0	n.a.	n.a.
	σ*_jkp_*	185.2 (73.4–1169.4)	n.a.	n.a.
	ICC*_jkp_*	0.34 (0.013–0.76)	n.a.	n.a.
*Y_k_* = *U*_max_	Optimal model:	M1a: *U*_max_ = α*_kp_* + FL*_j_* + FR*_j_* + *a_jkp_* + ϵ*_ijkp_*		
	α*_kp_*	−1.53	0.32	<0.001
	FL*_j_*	0.0041	0.00083	<0.001
	FR*_j_*	0.29	0.068	<0.001
	σ*_akp_*	0.029	n.a.	n.a.
	σ*_jkp_*	0.12 (0.03–0.28)	n.a.	n.a.
	ICC*_jkp_*	0.051 (0.011–0.55)	n.a.	n.a.
*Y_k_* = *U*_inst_	Optimal model:	M1a: *U*_inst_ = α*_kp_* + FL*_j_* + FR*_j_* + *a_jkp_* + ϵ*_ijkp_*		
	α*_kp_*	−2.60	0.47	<0.001
	FL*_j_*	0.00087	0.0011	0.44
	FR*_j_*	0.24	0.10	0.015
	σ*_akp_*	0.052	n.a.	n.a.
	σ*_jkp_*	0.26 (0.19–0.46)	n.a.	n.a.
	ICC*_jkp_*	0.037 (0.013–0.068)	n.a.	n.a.
*Y_k_* = AR_day_	Optimal model:	M1: AR_day_ = α*_kp_* + FL*_j_* + a*_jkp_* + ϵ*_ijkp_*		
	α*_kp_*	−582.0	418.5	0.17
	FL*_j_*	5.11	2.59	0.0497
	σ*_akp_*	109.9	n.a.	n.a.
	σ*_jkp_*	111.4 (53.5–998.1)	n.a.	n.a.
	ICC*_jkp_*	0.49 (0.01–0.81)	n.a.	n.a.

Parameter estimates and associated standard errors (where available) are given. Medians are given for ${\sigma_{jkp}}^{2}$ and ICC*_jkp_*, with the minimum and maximum in parentheses. For all four models, it is assumed that $a_{jkp}\tilde{}N(0,{\sigma_{akp}}^{2})$ and $\epsilon_{ijkp}\tilde{}N(0,{\sigma_{jkp}}^{2})$. *P*-values are obtained from likelihood ratio tests comparing the optimal model with a nested model excluding the respective parameter.

## Discussion

By combining laboratory and field techniques, we tested hypotheses derived from conceptual models (Blake, 1983; Careau *et al.*, 2008; Pörtner, 2010) predicting that individual metabolism and morphology correlate with *in situ* activity. Surprisingly, we found that individual metabolism and activity are independent, indicating that a strong link between these traits is not universally present. As predicted, we found that individual morphological variation explained variation in activity measures, indicating that morphological variation is a determinant of locomotion patterns.

### Metabolism and activity

Defining aerobic performance and being highly dependent on ambient temperature, aerobic metabolism is used as both an indicator and a predictor in relationship to conservation of fish species and their responses to anthropogenic stressors, including rising temperatures mediated by climate change (Horodysky *et al.*, 2015; Schulte, 2015), particularly through the OCLTT hypothesis (Pörtner, 2010). Additionally, metabolism has been recognized in a series of studies as a possible mechanistic link between environmental conditions, life history and behaviour (Brown, 2004; Careau *et al.*, 2008; Biro and Stamps, 2010; Horodysky *et al.*, 2015), although the topic remains debated (Halsey *et al.*, 2015; Mathot and Dingemanse, 2015). The OCLTT hypothesis implies that critical performances, such as growth and locomotion, are causally linked with aerobic scope. It is therefore conceivable that inter-individual variation in AMS should be correlated with individual activity. The present study, however, found no evidence of such correlations. Thus, in alignment with recent studies (Clark *et al.*, 2013; Ern *et al.*, 2014; Norin *et al.*, 2014), this indirect examination of the OCLTT hypothesis suggests that assumptions may not be fulfilled in the wild. While it remains unknown whether temperature-induced variation in AMS corresponding to the phenotypic variation observed in the present study will affect activity patterns, our study suggests that care should be taken if attempting to predict the performance of fish species (e.g. in relationship to climate change) from simple metrics of metabolism.

Standard metabolic rate (and equivalents) is the most-studied aspect of vertebrate metabolism (Careau *et al.*, 2008), and previous laboratory studies have found positive correlations between resting metabolic rate and behavioural parameters such as aggression, dominance and boldness in a number of taxa (reviewed by Biro and Stamps, 2010 and Mathot and Dingemanse, 2015). Although empirical studies on correlations between SMR and activity in fish are scarce, a single study (Farwell and McLaughlin, 2009) was identified by Biro and Stamps (2010) and Mathot and Dingemanse (2015). Using recently emerged brook charr (*Salvelinus fontinalis*), Farwell and McLaughlin (2009) found no correlation between SMR and activity measured as time spent moving. In contrast, Watz *et al.* (2015) found correlations between individual resting metabolic rate and swimming activity in brown trout (*Salmo trutta*) tested in an indoor stream channel. Likewise, Myles-Gonzalez *et al.* (2015) tested round goby (*Neogobius melanostomus*) in an artificial flume and found that more active fish also exhibit elevated resting metabolic rate. Interestingly, it has been suggested that environmental stressors such as temperature and hypoxia can reveal, mask and modulate the covariation of physiological and behavioural traits (Killen *et al.*, 2013, 2012a). Thus, although SMR is potentially correlated with some behavioural parameters measured in laboratory settings, the link between SMR and activity in non-stressed conditions can be weak or non-existent, as found in the present study.

Critical and optimal swimming speeds are measures of fish swimming performance obtained using laboratory protocols that involve forced swimming (e.g. Claireaux *et al.* 2006; Tudorache *et al.* 2008). Although the mechanisms are not fully understood, these performance measures are often linked to variation in metabolism (Claireaux *et al.*, 2005; Arnott *et al.*, 2006; Binning *et al.*, 2015). For instance, Binning *et al.* (2015) found MMR to be the best overall predictor of individual swimming performance. These findings could be important for the present study because forced laboratory measures of MMR may correlate positively with MMR measured in spontaneously active fish (Svendsen *et al.*, 2014). A correlation between *U*_max_ and MMR (and/or AMS) was therefore expected but not found in the present study. Furthermore, Claireaux *et al.* (2006) found that European sea bass (*Dicentrarchus labrax*) reach their maximal aerobic capacity at swimming speeds near the critical swimming speed and that metabolism when swimming at optimal swimming speed represents a consistent percentage of MMR. Thus, under the assumption often used in the literature that free-ranging fish swim at or near optimal swimming speed during routine swimming (Videler, 1993; Claireaux *et al.*, 2006; Tudorache *et al.*, 2011; Svendsen *et al.*, 2015), a correlation between *U*_inst_ and MMR and/or AMS was expected but not found in the present study. However, when determining maximal and critical swimming speeds, fish are typically forced to swim until exhaustion. Although these measures give insights into the maximal capacity of the fish, they may not be biologically relevant. For example, it is currently uncertain to what extent fish use their full AMS spontaneously and in natural settings (Lucas *et al.*, 1993; Murchie *et al.*, 2011; Genz *et al.*, 2013). Likewise, the assumed relationship between optimal swimming speed and spontaneous swimming speed of fish in the wild has yet to be confirmed (Claireaux *et al.*, 2006; Tudorache *et al.*, 2011; Svendsen *et al.*, 2015). Finally, the correlation between AMS and swimming performance is not consistent in all fish species (Anttila *et al.*, 2014; Svendsen *et al.*, 2015) and may not be present in European perch.

The complete lack of correlations between metabolism and activity levels was surprising. Besides the possibility that no correlation exists, there are potential sources of error that could enshroud existing correlations. Apart from the inherent uncertainties in both the metabolic measurements and the activity metrics, the combination of laboratory and *in situ* measurements could introduce context-specific biases. For instance, individual personality might affect the measurement accuracy in metabolic studies through individual differences in reactions to being confined in a respirometry chamber (Careau *et al.*, 2008). Further studies are required to examine stress levels of animals in respirometer chambers and test the hypothesis that disparate behavioural phenotypes react differently to respirometer confinement. Additionally, the translocation of the fish from lake to laboratory and back again could induce stress responses extending the recovery periods and altering both metabolic and behavioural phenotypes. However, the activity levels and spatial distribution of tagged European perch in the present study were comparable (H. Baktoft, unpublished data) to those of conspecifics tagged and immediately returned to Lake Gosmer as part of another study (Jacobsen *et al.*, 2014), suggesting that behaviours were unaffected by the translocation and laboratory tests.

### Morphology and activity

Individual FL was positively correlated with *A*_day_, AR_day_ and *U*_max_, but not with *U*_inst_. The positive correlations between FL and *A*_day_, AR_day_ and *U*_max_ were expected because larger fish generally use larger areas and are able to swim faster. Following this rationale, a correlation between FL and *U*_inst_ was also expected. European perch is a schooling species (Thorpe, 1977), and they often aggregate in foraging groups (Eklöv, 1992), suggesting that the swimming speed of the tagged fish could have been influenced by other school members and not determined solely by individual traits. However, when acknowledging slight differences in body lengths, other studies have found comparable speeds of actively swimming European perch (Zamora and Moreno-Amich, 2002; Linløkken *et al.*, 2010), indicating that *U*_inst_ measured in the present study is within a credible range.

Fineness ratio explained significant proportions of the variation in both *U*_max_ and *U*_inst_. Previous theoretical and empirical studies have shown that higher FR (or equivalent measures) up to a given threshold are generally associated with lower swimming costs (Blake, 1983; Boily and Magnan, 2002; Ohlberger *et al.*, 2006; Chung, 2009). A previous field study linking detailed fish behaviour with morphological characteristics found correlations between a composite morphometric measure comparable to FR and mean speed and mean daily distance in largemouth bass (Hanson *et al.*, 2007). The present study adds empirical field evidence emphasizing the biological relevance of individual morphological differences in relation to swimming speeds. Interestingly, recent studies have concluded that FR is not a strong predictor of swimming performance (Fisher and Hogan, 2007; Hendry *et al.*, 2011; Dalziel and Schulte, 2012; Walker *et al.*, 2013; Binning *et al.*, 2015); nonetheless, we found that FR predicts variation in both *U*_max_ and *U*_inst_, perhaps indicating that spontaneous activity is not always predicted by maximal activity measured in the laboratory.

Although the prospect of a purely physical explanation (i.e. the hydrodynamic effects of FR affecting swimming costs) of parts of the individual variability in activity is alluring, this correlation could include biological components as well. For instance, European perch morphology is known to be plastic and correlated with habitat structure, feeding mode and temperature (Olsson and Eklöv, 2005; Rowiński *et al.*, 2015). Generally, deep-bodied (i.e. lower FR) and thus more manoeuvrable European perch are associated with the benthic niche, whereas slender European perch (i.e. higher FR) are associated with the pelagic (Hjelm *et al.*, 2000; Svanbäck and Eklöv, 2004). This differentiated niche association *per se* could entail behavioural differences influencing activity levels. Thus, the effects of FR on activity levels might not be directly exerted through hydrodynamic effects, but could instead operate indirectly through evolutionary processes shaping the morphology–niche correlation. However, irrespective of the causal mechanism driving the correlations between FR and activity, it is interesting that a simple morphological metric can explain a significant amount of phenotypic behavioural variation in the wild.

### Study assumptions

The present study relies on several assumptions, including the following.

First, the tagged European perch behaved naturally, i.e. as untagged conspecifics. This is a fundamental assumption in most studies using telemetry but impossible to validate in the present study.

Second, measured metabolic rates reflected the amount of adenosine triphosphate (ATP) generated aerobically (Salin *et al.*, 2015) and were repeatable and temporally consistent. The latter could not be verified owing to time restrictions set by the transmitter battery life, but previous studies suggest that this is a valid assumption (Nespolo and Franco, 2007; Maciak and Konarzewski, 2010; Norin and Malte, 2011; Svendsen *et al.*, 2014), although repeatability in tagged and translocated fish has not been tested. Moreover, repeatability of the individual metabolic traits can be influenced by environmental factors, such as temperature and hypoxia (Careau *et al.*, 2014; Norin *et al.*, 2015), possibly affecting our findings.

Third, the activity metrics obtained using the telemetry system truthfully reflected fish activity patterns and were relevant in terms of fish balancing their energy budgets in an adaptive fashion (Mathot and Dingemanse, 2015). The validity of data produced by the system has previously been assessed by standardized tests using stationary transmitters and by towing transmitters to mimic swimming fish (Fig. 2). These tests showed very good performance in terms of efficiency, accuracy and precision, and the tow tracks showed very good alignment with true tracks obtained using a differential GPS (Baktoft *et al.*, 2015). However, the estimation accuracy of activity metrics (especially *U*_inst_ and *U*_max_) might have been influenced by the transmitter burst intervals (30 s) because any movement beyond straight-line distance between consecutive positions cannot be detected by the system. The selected burst interval was chosen as a compromise between battery life expectancy (i.e. longevity of the study period) and transmitter size.

Fourth, the difference in water temperature between the laboratory facility and the study lake could potentially enshroud links between metabolism and activity. However, water temperature in the laboratory had to be decided *a priori* and kept fixed for the duration of the laboratory work. Moreover, if shifts in temperature well within the natural range of a species (Thorpe, 1977) dramatically alter linkage between metabolism and activity, the entire concept of causal or mechanistic links between these parameters seems tenuous, particularly in relation to animal conservation and evolutionary patterns.

Fifth and finally, it should be noted that the results in the present study are based on a relatively low sample size, partly owing to predation events and transmitter failure. Therefore, care should be taken when interpreting the results. However, even when correcting for pseudoreplication by using a mixed model approach, several of the findings were highly significant, adding credibility to the results.

### Conclusions

The present study suggests that although metabolism is closely related to energy use in individual animals, a direct link to volitional activity is missing. Thus, we found no support for the overarching hypothesis that individual metabolic traits influence individual activity, suggesting that causal links between metabolism and activity derived from the OCLTT hypothesis and the allocation and performance models are not always present in natural settings. In contrast, we found several indications that fish size and morphology are correlated with fish activity, suggesting a stronger link between these factors. Although the conceptual models discussed here represent powerful tools to understand the intricate links between metabolism, environmental variation and animal performance, this study adds to the body of evidence that animal activity patterns are highly complex and variable and are difficult to capture and explain using relatively simple metrics. The complex nature of animal activity patterns remains a challenge for studies providing data for science-based strategies related to management and conservation.

## Funding

This work was supported by the Danish Rod and Net Fish License Funds and the Foundation for Science and Technology (FCT) in Portugal [SFRH/BPD/89473/2012] to J.C.S.
